# Editorial of Special Issue “Synthesis and Molecular Applications of Metal-Organic Frameworks (MOFs)”

**DOI:** 10.3390/ijms24097857

**Published:** 2023-04-26

**Authors:** Mikhail Khrizanforov

**Affiliations:** Arbuzov Institute of Organic and Physical Chemistry, FRC Kazan Scientific Center of RAS, Arbuzov Str. 8, 420088 Kazan, Russia; khrizanforov@gmail.com; Tel.: +7-(843)-273-22-93; Fax: +7-(843)-273-22-53

This Special Issue is dedicated to exploring various approaches and techniques for the preparation and modification of metal–organic frameworks (MOFs), as well as their applications, with a specific focus on molecular research [1,2,3,4]. Over the past few years, research in this field has continued to evolve and garner significant attention. This is not surprising because MOFs have a wide range of applications in diverse fields such as sorption materials, catalysis, semiconductors, storage, fuel cells, compounds separation, and solid-state sensors, among others.

The aim of this Special Issue on “Synthesis and Molecular Applications of Metal-Organic Frameworks (MOFs)” is to provide an open platform for researchers to share their investigations and findings in this innovative field. Overall, this Special Issue strives to foster a collaborative and productive exchange of scientific ideas and knowledge and advance the field of MOF synthesis and applications.

Timofeeva M.N. et al. discuss the potential of zeolitic imidazolate frameworks (ZIFs) for acid–base catalysis [1]. The authors aim to provide insights into the structure-property-activity relationship of ZIFs for acid–base catalysis. They analyze published articles and supporting information from journals to gain a better understanding of the topic. The authors discuss the advantages and disadvantages of using ZIFs for acid–base catalysis. The advantages are that ZIFs have high thermal stability, which makes them suitable for high-temperature reactions and a high surface area, which allows for more active sites and increased reactivity. The review notes that the complexity of ZIF synthesis limits their practical application. Additionally, their stability in acidic or basic environments can be an issue. The authors also discuss the different types of ZIFs and their properties. They explain how the structure of ZIFs affects their catalytic activity and selectivity. Overall, the authors conclude that ZIFs have great potential for acid–base catalysis due to their unique properties and structure. However, further research is needed to fully understand their behavior in different environments and to optimize their synthesis for practical applications.

The Milyukov V.A. team has published a study on the formation of a 2D coordination polymer based on 1,1′-ferrocene-diyl-bis(*H*-phosphinic acid)) with Sm(III) nodes [2]. The study investigated the role of the phosphinate group in the formation of this polymer. The authors investigate coordination polymers, comparing their electrochemical properties to those of metal–organic frameworks and analyzing X-ray diffraction and Mössbauer studies. The results showed that the phosphinate group plays a crucial role in the formation of this polymer [2]. The researchers found that it acts as a bridging ligand between Sm(III) nodes, forming a 2D network structure. This structure is stable and can be used for various applications in molecular sciences. The X-ray structural analysis revealed that the polymer has a layered structure with Sm(III) nodes connected by phosphinate groups. The electrochemical studies showed that the polymer has good conductivity and can be used as an electrode material. The Mössbauer spectroscopy analysis revealed that Sm(III) ions are presented in two different environments within the polymer. This indicates that there is some degree of disorder within the structure. Overall, this study provides valuable insights into the formation and properties of 2D coordination polymers with Sm(III) nodes. The use of phosphinate groups as bridging ligands offers several advantages, including stability and good conductivity.

Ajibade P.A. and Oloyede S.O. have discussed the synthesis of metal–organic frameworks quantum dots composites (MOFs@QDs) as sensors for endocrine-disrupting chemicals [3]. The authors note that while various sensors have been developed to detect bisphenol A, the use of composite materials as sensors for this chemical has not been explored. As a result, they propose the development of MOFs@QDs for this purpose. The article provides a review of synthetic techniques for quantum dots in aqueous media and their use in detecting endocrine-disrupting chemicals. The authors examine the unique electronic properties and two-dimensional carbon structure of graphene oxide, which can be incorporated into metal–organic frameworks to develop binary quantum dots/metal–organic frameworks for probing endocrine-disrupting chemicals. The article discusses the method of synthesis, optoelectronic properties, and applications of metal–organic frameworks and quantum dots. The authors note that MOFs@QDs offer several advantages over traditional sensors, including high sensitivity, selectivity, and stability. They also discuss potential applications for these composites in detecting and monitoring endocrine-disrupting chemicals in various settings. The authors acknowledge that there are some limitations to their proposed approach, including challenges related to scalability and cost-effectiveness. In conclusion, the authors propose the development of MOFs@QDs as a promising approach for detecting endocrine-disrupting chemicals such as bisphenol A. While there are some limitations to this approach, they believe that further research can address these challenges and lead to new applications for these composites in environmental monitoring and other fields.

The review of Saura-Sanmartin A. [4] explores the use of photoresponsive metal–organic frameworks (MOFs) as adjustable scaffolds in reticular chemistry. The easy and remote switching of light makes it an ideal stimulus for a large number of applications, including the preparation of photoresponsive materials. MOFs are ideal scaffolds for light-responsive ligands due to their structural complexity. The integration of photoresponsive organic ligands inside MOF crystalline arrays can prepare enhanced functional materials. These materials have potential applications in areas such as sensing, drug delivery, and catalysis. The use of MOFs allows for precise control over the properties and functions of these materials. However, there are still challenges to be addressed, such as the limited availability of suitable photoresponsive ligands and the need for more efficient methods for their incorporation into MOFs.

The future outlook for this research area is promising, with potential advancements in areas such as optoelectronics and energy storage. Overall, this review provides a comprehensive overview of the preparation, properties, and applications of photoresponsive MOFs and highlights their potential as adjustable scaffolds in reticular chemistry.

## References

[1] Timofeeva M.N., Panchenko V.N., Jhung S.H. (2023). Insights into the Structure–Property–Activity Relationship of Zeolitic Imidazolate Frameworks for Acid–Base Catalysis. Int. J. Mol. Sci..

[2] Shekurov R.P., Khrizanforov M.N., Zagidullin A.A., Zinnatullin A.L., Kholin K.V., Ivshin K.A., Gerasimova T.P., Sirazieva A.R., Kataeva O.N., Vagizov F.G. (2022). The Phosphinate Group in the Formation of 2D Coordination Polymer with Sm(III) Nodes: X-ray Structural, Electrochemical and Mössbauer Study. Int. J. Mol. Sci..

[3] Ajibade P.A., Oloyede S.O. (2022). Synthesis of Metal–Organic Frameworks Quantum Dots Composites as Sensors for Endocrine-Disrupting Chemicals. Int. J. Mol. Sci..

[4] Saura-Sanmartin A. (2022). Photoresponsive Metal-Organic Frameworks as Adjustable Scaffolds in Reticular Chemistry. Int. J. Mol. Sci..

